# Supplementary material for: Unselected Population Genetic Testing for Personalised Ovarian Cancer Risk Prediction: A Qualitative Study Using Semi-Structured Interviews
Source: Diagnostics (Basel). 2022 Apr 19;12(5):1028. doi: 10.3390/diagnostics12051028 (PMC9139231; doi:10.3390/diagnostics12051028)
Supplement: Supplementary file 1 [file diagnostics-12-01028-s001.zip › diagnostics-1675183-supplementary.pdf]

PROMISE FEASIBILITY STUDY

Supplementary Material: Topic Guide
Unselected Population Genetic Testing for Personalised Ovarian Cancer Risk Prediction: a Qualitative Study Using Semi-Structured Interviews

Introduction

- Introduce self, research study funded by CRUK and The Eve Appeal charity.
- Purpose of the research.
- Introduce audio recorder.
- Stress all identifiable information will be kept confidential, but anonymised quotes may be published/presented.

Interview

- Family composition
  Q: Who lives at home? Tell me about your relationships?
  P: Partner, children, parents, siblings, ages
- Support network
  Q: Tell me about people who are important to you that give you support/advice when you need it.
  P: Friends, family, support groups, religion
- Occupation
  Q: How would you describe your occupation?
  P: Full time, part time, paid, unpaid
- Hobbies
  Q: How do you fill your spare time? What do you do in your spare time?

- Importance of health to the individual.
  Q: *How important is being healthy to you?*
- Motivations for staying healthy.
  Q: What motivates you to stay healthy? *Why is it important?*
  P: Family, friends, duty to self, cultural expectations

- Previous knowledge of PGT.
  Q: *Were you aware of genetic testing and risk assessment in women who have not had OC? Where did you get your information from?*
  P: health professionals, reading, media
- Initial thoughts on hearing about PGT.
  Q: *What were your initial thoughts on hearing about this? Why did you feel this way?*

- Reasons PGT/OC risk assessment acceptable
  Q: Why do you think you chose to undergo genetic testing?
  P: OC worry, desire to stay healthy, altruism, curiosity, know someone who has had a genetic test, family history
- Reasons PGT/OC risk assessment not acceptable
  Q: Were there any aspects of genetic testing that you found unacceptable?
  P: insurance implications, employability, change in family dynamics, stigma
- Does acceptability change with age
  Q: How might your decision have been different if you were older/younger?
- Reaction of family/friends in relation to her having PGT/OC risk assessment.
  Q: *Did you discuss your decision with family/friends? What did they think of you having a genetic test?*
- Ease of coming to a decision
  Q: *How easy was it to come to a decision?*
  P: What made it easy/difficult

- Effect of results on the individual's health
  Q: How has your life changed after learning the results of your genetic test and lifetime risk of ovarian cancer?"
  P: Ovarian cancer worry, *psychological health, impact of screening and prevention (intermediate-high risk only)*
- Effect of results on family, friends.
  Q: *How has having had a genetic test and learning about your lifetime risk of ovarian cancer affected your family/friends?*
- Change in lifestyle choices since results
  Q: *What lifestyle changes have you made, if any, since learning your results?*
  P: Smoking, drinking, exercise, diet, vitamins, herbal/complementary therapy
- Reason(s) for change in lifestyle choices.
  Q: *Why did you make these changes?"*
  P: Family, friends, cultural expectations

- Perceived gains of PGT and OC risk stratification.
  Q: *What do you think have been the benefits/positives of genetic testing and learning your lifetime risk of OC?*
- Perceived losses.
  Q: *What do you think have been the negatives/pitfalls?*

- Overall satisfaction with pre-test information prior to making the decision to undergo/not undergo PGT and OC risk assessment.
  Q: *Overall how satisfied are you with the online decision aid/telephone helpline/written information you received before you made your decision? In retrospect, is there anything you know now which you did not know before having the test but you wish you knew?*
  P: *Did the information received help make a decision, why did it help, what else helped*
- Regrets.
  Q: What regrets, if any, do you have about the choice you made? If you had the chance to make your decision again, would you still choose to be tested and learn your lifetime risk of ovarian cancer?
- Likelihood of future regret
  Q: *How do you think you will feel about your decision in a few months/years? Are there any circumstances which may make you regret your decision?*
  P: Reasons for regret
- Areas for improvement.
  Q: *How would you improve the information that is provided?*
- Advice she would give to someone trying to make a decision about undergoing PGT.
  Q: *What advice would you give to someone faced with having to make a similar choice as yourself?*

Final steps:<br>Thank the participant. Check whether they have remaining questions or comments about the topic<br>Reassurance about confidentiality and anonymity<br>PROMISE Feasibility Study contact details should they want further information

Q – question; P – probe; CRUK – Cancer Research UK; PGT – population genetic testing; OC – ovarian cancer.
